# Disturbance in Plasma Metabolic Profile in Different Types of Human Cytomegalovirus-Induced Liver Injury in Infants

**DOI:** 10.1038/s41598-017-16051-8

**Published:** 2017-11-16

**Authors:** Wei-Wei Li, Jin-Jun Shan, Li-Li Lin, Tong Xie, Li-Li He, Yan Yang, Shou-Chuan Wang

**Affiliations:** 1 Affiliated Hospital of Nanjing University of Chinese Medicine, Department of Pediatrics, Nanjing, 210023 China; 2 Jiangsu Key Laboratory of Pediatric Respiratory Disease, Institute of Pediatics, Nanjing University of Chinese Medicine, Nanjing, 210023 China; 3 Beijing Children’s Hospital Affiliated to Capital Medical University, TCM Department, Beijing, 100045 China; 4 Affiliated Hospital of Integrated Traditional Chinese and Western Medicine, Nanjing University of Chinese Medicine, Nanjing, 210023 China

## Abstract

Human cytomegalovirus (HCMV) infection in infants is a global problem and the liver is a target organ of HCMV invasion. However, the mechanism by which HCMV causes different types of liver injury is unclear, and there are many difficulties in the differential diagnosis of HCMV infantile cholestatic hepatopathy (ICH) and extrahepatic biliary atresia (EHBA). We established a non-targeted gas chromatography-mass spectrometry metabolomics method in conjunction with orthogonal partial least squares-discriminate analysis based on 127 plasma samples from healthy controls, and patients with HCMV infantile hepatitis, HCMV ICH, and HCMV EHBA to explore the metabolite profile of different types of HCMV-induced liver injury. Twenty-nine metabolites related to multiple amino acid metabolism disorder, nitrogen metabolism and energy metabolism were identified. Carbamic acid, glutamate, L-aspartic acid, L-homoserine, and noradrenaline for HCMV ICH *vs*. HCMV EHBA were screened as potential biomarkers and showed excellent discriminant performance. These results not only revealed the potential pathogenesis of HCMV-induced liver injury, but also provided a feasible diagnostic tool for distinguishing EHBA from ICH.

## Introduction

As one of the largest members of the herpesvirus family, human cytomegalovirus (HCMV) is closely linked to its natural host, humans. HCMV is ubiquitous, and the prevalence of congenital HCMV has been reported as 0.2–2% in the developed world and 0.6–6.1% in the developing countries based on limited studies^1^. Liver damage is common in congenital and perinatal HCMV infection. The clinical manifestations of HCMV- induced liver injury vary greatly between patients, ranging from mildly symptomatic disease (e.g. mild hepatomegaly or raised levels of transaminases) to moderate and severe disease (e.g. hepatitis, cholestatic hepatopathy, hepatomegaly, and even cirrhosis)^2–4^.

However, the aetiology and pathogenesis of HCMV-induced liver injury are not well-understood. Some studies suggested detecting HCMV-DNA in liver tissue, which indicates that the replication of HCMV can cause direct damage to the liver^5,6^. However, HCMV-DNA was not detected in liver tissue in other studies^7,8^. Thus, it remains controversial whether the HCMV-DNA represents a carry-over effect from peripheral leukocytes. This leads to the hypothesis that persistent liver injury occurs because of an ongoing immunologic response to HCMV infection^8^.

Additionally, the clinical manifestations of HCMV-induced liver injury are diverse, making clinical diagnosis difficult. Extrahepatic biliary atresia (EHBA) is a common disease in paediatric surgery that can cause liver damage and even cirrhosis. EHBA has also been reported to be related to HCMV^5,7^. The clinical presentations of EHBA and infantile cholestatic hepatopathy (ICH) are similar. In both conditions, jaundice, hypopigmented stools, dark urine and hepatosplenomegaly develop within the first 3 month of life. Endoscopic retrograde cholangio-pancreatography and liver tissue pathology are the gold standards for distinguishing between EHBA and ICH, but both of these tests are invasive. However, EHBA should be differentiated from ICH as soon as possible, as a good outcome for EHBA infants depends on the early diagnosis and timely kasai portoenterostomy^9^. Therefore, new techniques are needed for the differential diagnosis of ICH and EHBA, and the mechanisms by which HCMV causes different types of liver injury should be determined.

Metabolomics, a branch of “omics” research, involves the assessment of metabolites in biological samples and defines the information closest to the phenotype of the biological system^10^. In recent years, with the combination of different metabolomics detection methods^11,12^, the update of methodology^13,14^, and the combination with other omics technology^15,16^, analysis of metabolites in biological samples is no longer a problem. Metabolic profiling has the potential to identify novel biomarkers to facilitate early diagnosis and prognosis of HCMV-induced liver injury. Recent studies examined metabolic changes in HCMV primary infection in pregnancy and neonates^17,18^. A series of cell metabolomic studies have also evaluated the changes in cellular metabolism caused by HCMV infection, which involve the metabolism of glucose and glutamine^19–22^. However, less attention has been given to metabolomics in liver injury caused by HCMV infection.

In the present study, we analyzed the endogenous metabolites in plasma samples from infants with HCMV-induced liver injury using untargeted gas chromatography-mass spectrometry (GC-MS). Orthogonal partial least squares-discriminate analysis (OPLS-DA) was applied to analyse the differences between HCMV infantile hepatitis (IH), HCMV ICH, HCMV EHBA and normal control (NC) subjects.

## Results

### Characteristics of study subjects

A total of 127 participants were recruited, including 22 infants with HCMV IH, 39 infants with HCMV ICH, 26 infants with HCMV EHBA and 40 NC. The baseline clinical characteristics of the patients are shown in Table 1. Serum aspartate transaminase, total bilirubin, direct bilirubin, total bile acid, gamma-glutamyltransferase, alkaline phosphatase, and prothrombin time significantly differed between the three subgroups of infants with HCMV-induced liver injury (**P < **0.05). The age, BMI, and GCV use of the three subgroups are also statistically different (**P < **0.05). The roles of these cofounding factors need further discussion.

**Table 1:** Baseline characteristics in three HCMV induced liver injury sub groups of the study. Values are given as mean ± SD or number of individuals (%). ^a^P value of chi-square test. ^b^P value of Kruskal–Wallis test. ^c^P value of Dunn’s post hoc test.

Variables	HCMV IH	HCMV ICH	HCMV EHBA	P value^a^	IH *vs*. ICH^c^	IH *vs*. EHBA^c^	ICH *vs*. EHBA^c^
	(n = 22)	(n = 39)	(n = 26)				
Gender(female,%)	5(22.7%)	10(25.6%)	10(38.5%)	0.412466			
GCV use,%	12(54.5%)	27(69.2%)	8(30.8%)	0.00959			
				P value^b^			
Age,month	3.29 ± 1.69	2.38 ± 0.94	3.29 ± 2.03	0.024297	0.017954	0.611361	0.034049
BMI	17.83 ± 2.68	15.46 ± 2.70	16.33 ± 3.59	0.019104	0.014970	0.144924	0.312676
ALT,U/L	142.45 ± 133.92	175.66 ± 145.62	197.45 ± 156.12	0.176783	0.241394	0.054314	0.441319
AST,U/L	112.78 ± 100.91	246.68 ± 267.23	278.84 ± 150.89	0.000030	0.001060	0.000013	0.059016
TBIL,umol/L	11.16 ± 7.31	133.66 ± 85.87	162.42 ± 71.43	6.18E-12	1.17E-10	3.26E-09	0.023635
DBIL,umol/L	3.14 ± 2.57	81.37 ± 65.19	104.51 ± 48.10	2.49E-12	1.17E-10	3.26E-09	0.003823
TBA,umol/L	23.95 ± 30.85	102.20 ± 85.02	124.02 ± 52.82	3.69E-09	8.23E-08	1.39E-07	0.027206
GGT,U/L	110.54 ± 77.58	199.16 ± 221.16	363.48 ± 458.25	0.026581	0.028307	0.01968	0.252247
ALP,U/L	291.59 ± 132.70	595.58 ± 289.98	514.46 ± 250.45	0.000002	3.82E-07	0.000344	0.311748
ALB,g/L	37.27 ± 4.49	35.40 ± 5.40	36.98 ± 5.62	0.311954	0.326273	0.943610	0.138464
LDH,U/L	335.19 ± 75.96	401.61 ± 120.26	432.04 ± 352.01	0.094866	0.021247	0.360049	0.391435
PT,S	10.28 ± 0.96	11.89 ± 3.59	12.18 ± 3.29	0.010994	0.057359	0.002440	0.179165
WBC,10^9^/L	11.79 ± 6.01	11.92 ± 3.95	15.46 ± 12.34	0.427630	0.372848	0.220980	0.513357
NH3,umol/L	64.58 ± 17.18	82.74 ± 48.21	76.58 ± 40.84	0.100737	0.052500	0.493167	0.132249

### Untargeted metabolomics analysis revealed a clear distinction in metabolic profiles between subgroups of HCMV-induced liver injury and NC

**Figure 1:**
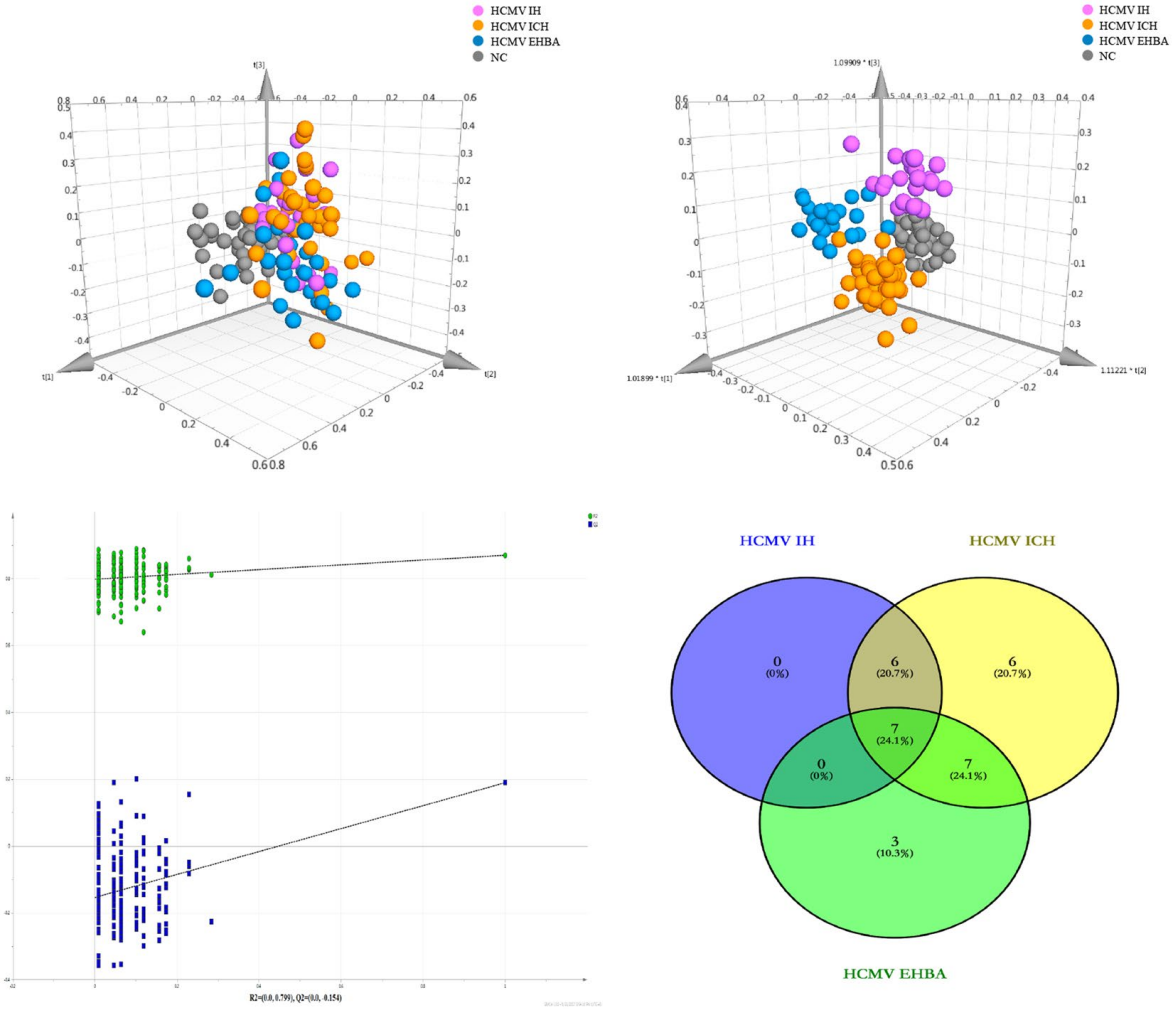
Differential metabolites and pathways related to HCMV induced liver injury. (**a**), PCA 3D Score plots of the HCMV IH group, HCMV ICH group, HCMV EHBA group and NC. (**b**), OPLS-DA 3D Score plots of the four groups. (**c**), Venn diagram of the differential metabolites in different HCMV induced liver injury groups compared with the NC. (**d**), the summary of aberrant pathways in HCMV induced liver injury, as analyzed by MetaboAnalyst 3.0.

We investigated the metabolite profiles of individual plasma samples by GC-MS. After alignment and normalisation of the data sets, we obtained a total of 674 metabolites detected (194 known and 480 unknown metabolites). In this study, principle component analysis (PCA) was first used to determine the general relationships between the four groups. PCA 3D score plots were provided for clustering display. As shown in Fig. 1(a), the disease subgroups and NC were clearly separated and the disease subgroups showed a separation trend, but the differentiation was not obvious. Subsequently, partial least square-discriminant analysis (PLS-DA) was performed to highlight the differences in metabolic profiling. As shown in Fig. 1(b), the PLS-DA 3D score plots revealed clear differentiation between the three subgroups of patients and control groups. Then the PLS-DA model was further validated using a permutation test (n = 200) to avoid overfitting (Fig. 1(c)). The OPLS-DA models of different subgroups *vs*. NC are shown in Supplementary Figure 1. Permutation tests of these models are also shown in Fig. S1. Specific data (R^2^Y and Q^2^Y values, R^2^ and Q^2^ values of permutation test) for these models are shown in Supplementary Table 1. These findings indicated that these models had good stability and repeatability, and could be exploited in subsequently differential metabolite search.

Using PCA, we also tested the role of confounding factors like age, BMI, and GCV use in separation of patients. As shown in Fig. S2, there was no separation trend among the groups. This suggests that the differentiation between the HCMV IH, HCMV ICH and HCMV EHBA groups is based on disease metabolite changes rather than on the roles of age, BMI, and GCV use in this study.

The reproducibility of data was assessed by using QC samples. As shown in Fig. S3, the PCA scores plot shows a clear cluster of the QC samples, indicating the high stability and reproducibility of the instrument. The relative standard deviations (RSD%) of peak area of internal standard 1,2-^13^C2-myristic acid was also calculated in the QC samples. The internal standard in QC samples have a RSD% of 6.2% means that the QC samples behaved stable for the duration of the run.

### Identification of differentially expressed metabolites in plasma of infants with HCMV-induced liver injury

For the 674 detected metabolites, we investigated differentially expressed metabolites (VIP > 1, **P** < 0.05 and FDR < 0.05). These endogenous metabolites were selected for further identification. Finally, 29 annotated differentially expressed metabolites between infants with HCMV-induced liver injury and NC above the threshold (log2 fold-change >1 or log2 fold-change <-1) are marked and presented in Supplementary Table 2. The heatmap of these 29 metabolites is shown in Fig. S4.

A Venn diagram was used to visualise the number of extremely different metabolites between HCMV IH, HCMV ICH, and HCMV EHBA groups and NC (Fig. 1(d)). Among the three subgroups of HCMV- induced liver injury, the difference between the HCMV IH group and NC was minimal, and the 13 differentially expressed metabolites in the HCMV IH versus NC were included in the 26 differentially expressed metabolites in HCMV ICH *vs*. NC. Norleucine, 3-hydroxy-L-proline, creatine, citraconic acid, palmitic acid, and myo-inositol were biomarkers of HCMV ICH infants compared to those of NC. Glutamate, citric acid, and D-galactose were significantly different in HCMV EHBA infants compared to those in NC (**P < **0.01). These results indicate that HCMV ICH and EHBA have specific metabolic characteristics.

**Figure 2:**
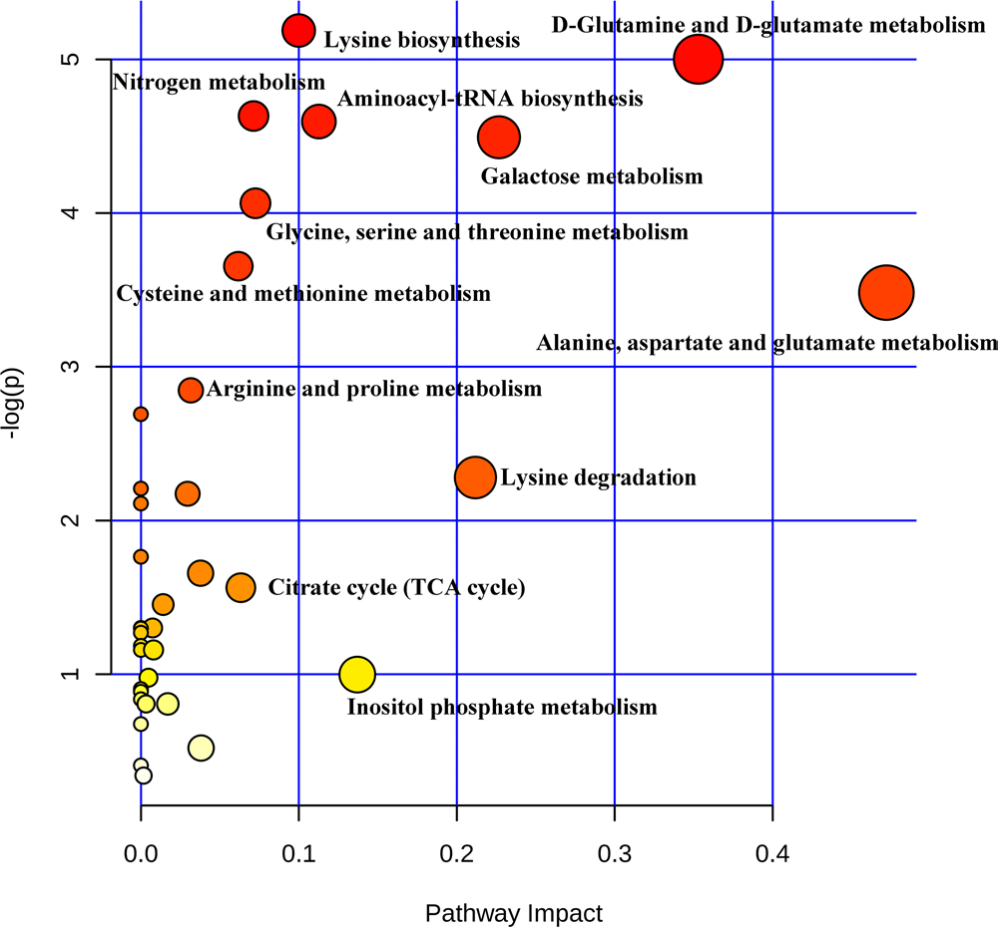
The summary of aberrant pathways in HCMV induced liver injury, as analyzed by MetaboAnalyst 3.0.

The biological pathways involved in the metabolism of these 29 metabolites were determined by enrichment analysis using MetaboAnalyst 3.0 (Fig. 2). All matched pathways were detected according to pathway impact values from pathway topology analysis (X-axis) and P values from the pathway enrichment analysis (Supplementary Table 3). Eight pathways were considered related to the development of HCMV- induced liver injury. These metabolic pathways are involved in a variety of amino acid and energy metabolism disorders.

### Diagnostic model for predicting HCMV EHBA

**Figure 3:**
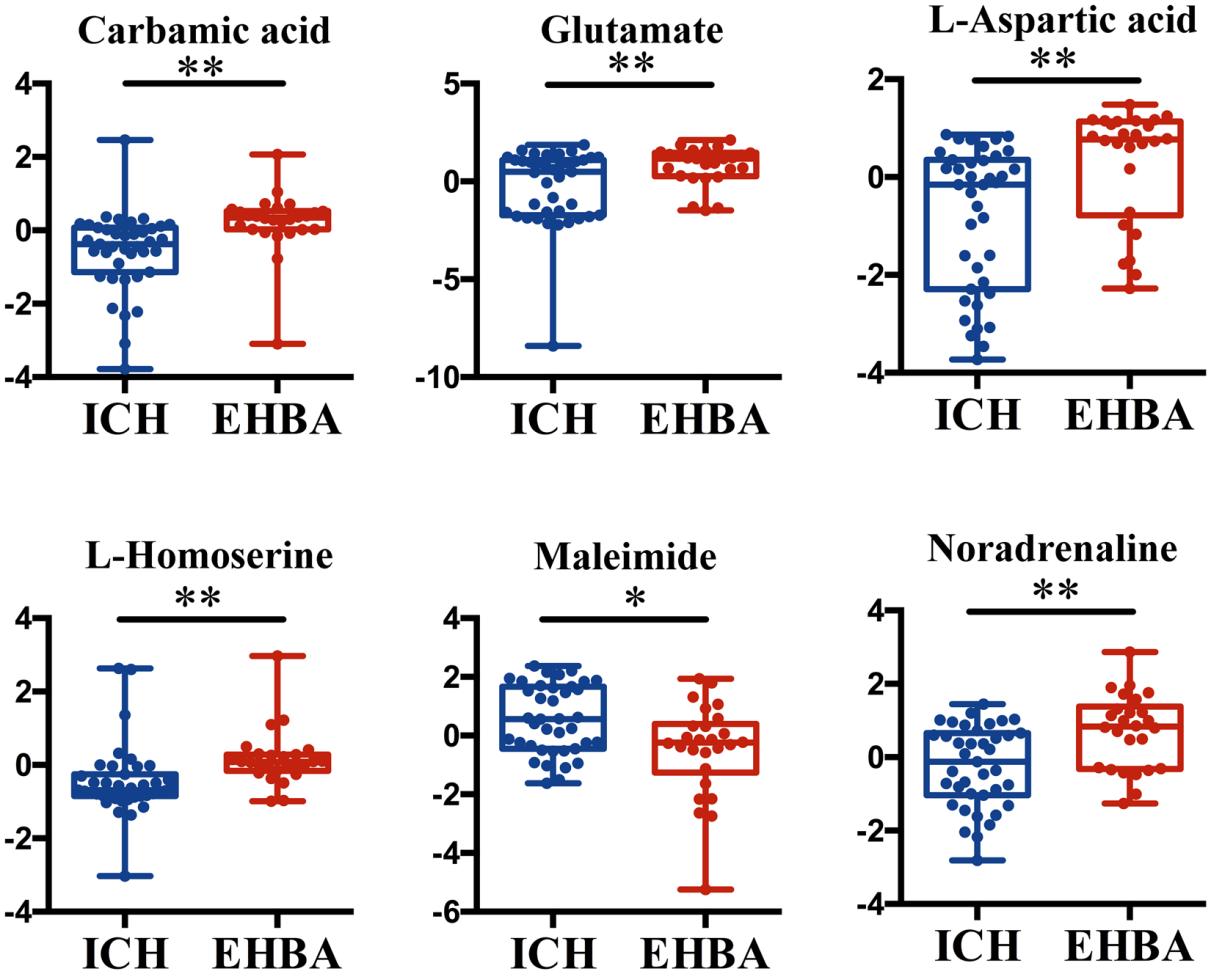
Boxplots showing up-regulated and down-regulated metabolites that could be used to differentiate HCMV ICH for HCMV EHBA.*P < 0.05, **P < 0.01.

To further investigate the differential metabolites between HCMV ICH and EHBA, PLS-DA mode was performed to distinguish between HCMV ICH, EHBA, and NC. Potential differential metabolites are shown in Supplementary Table 4. The metabolites exhibiting significant differences between HCMV ICH and EHBA groups are shown in Fig. 3.

**Figure 4:**
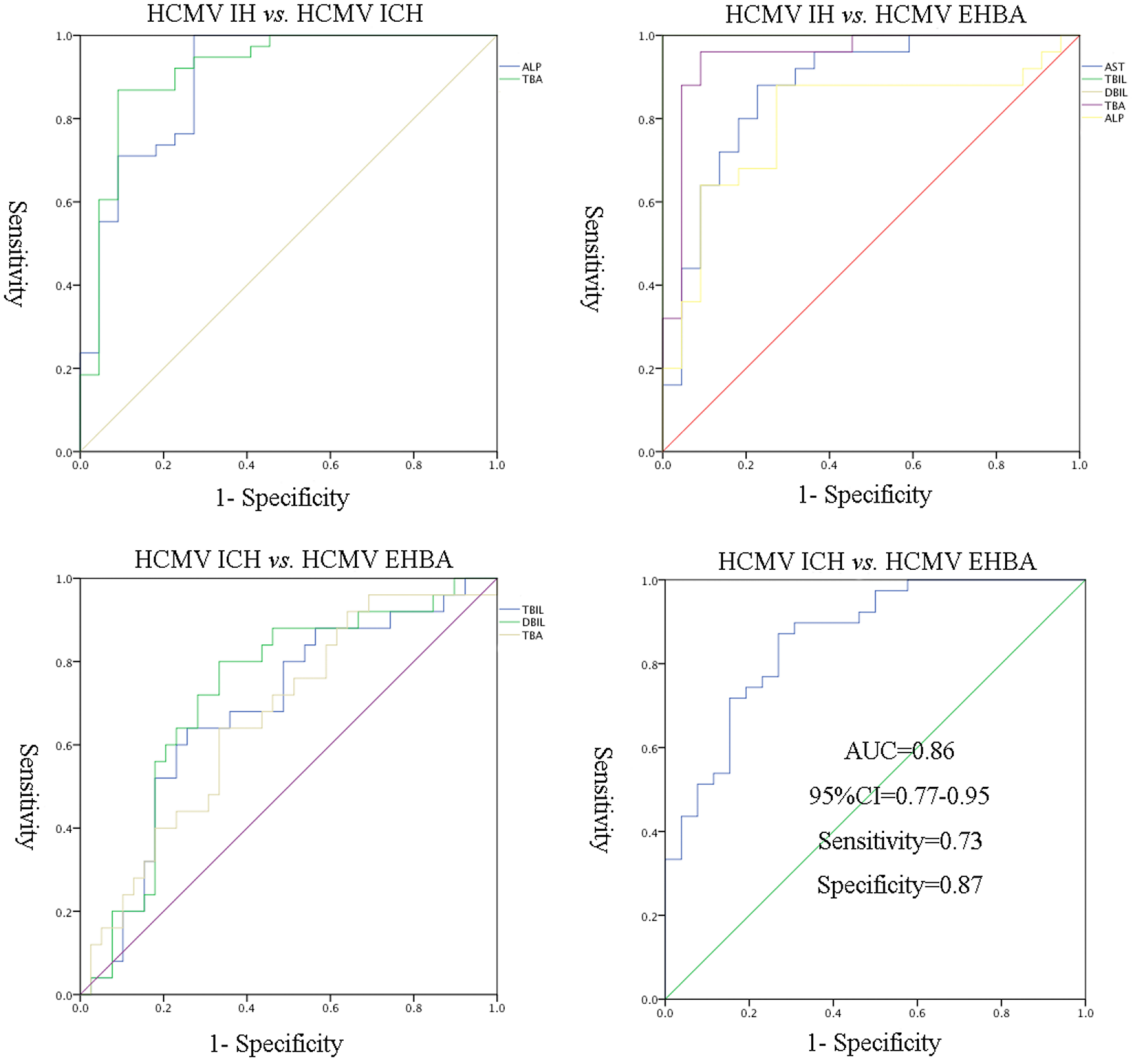
Receiver operating characteristics (ROC) curve model of clinical indicators and metabolites to discriminate HCMV IH, HCMV ICH from HCMV EHBA.

Based on Pearson correlation analysis, we analysed the correlation between the clinical indicators and metabolite markers, details is shown in Supplementary Table 5. The Pearson correlation coefficients ranges from 0.21 to 0.44, which means the clinical indicators and metabolite markers have little relevance. Then the receiver operating characteristics (ROC) presentations are made based on the logistic regression of differential clinical indicators among the three groups. As shown in Fig. 4(a,b), HCMV IH group *vs*. HCMV ICH group and HCMV IH group *vs*. HCMV EHBA group can be well differentiated by clinical indicators. However, as shown in Fig. 4(c), HCMV ICH group and HCMV EHBA group were poorly differentiated depending on clinical indicators. This is consistent with the current clinical situation that the differential diagnosis of these two diseases has been a difficult point. To improve the prediction of HCMV EHBA, a diagnostic model of more than one discriminatory metabolite was developed. Notably, as shown in Fig. 4(d), the combination of carbamic acid, glutamate, L-aspartic acid, L-homoserine, and noradrenaline enabled good prediction of HCMV EHBA (AUC = 0.86) with a sensitivity of 0.73 and specificity of 0.87. Thus, the distinctive signature with the five metabolites achieved a higher AUC value and increased the diagnostic performance of HCMV EHBA. Details of these ROC curves are shown in Supplementary Table 6.

## Discussion

**Figure 5:**
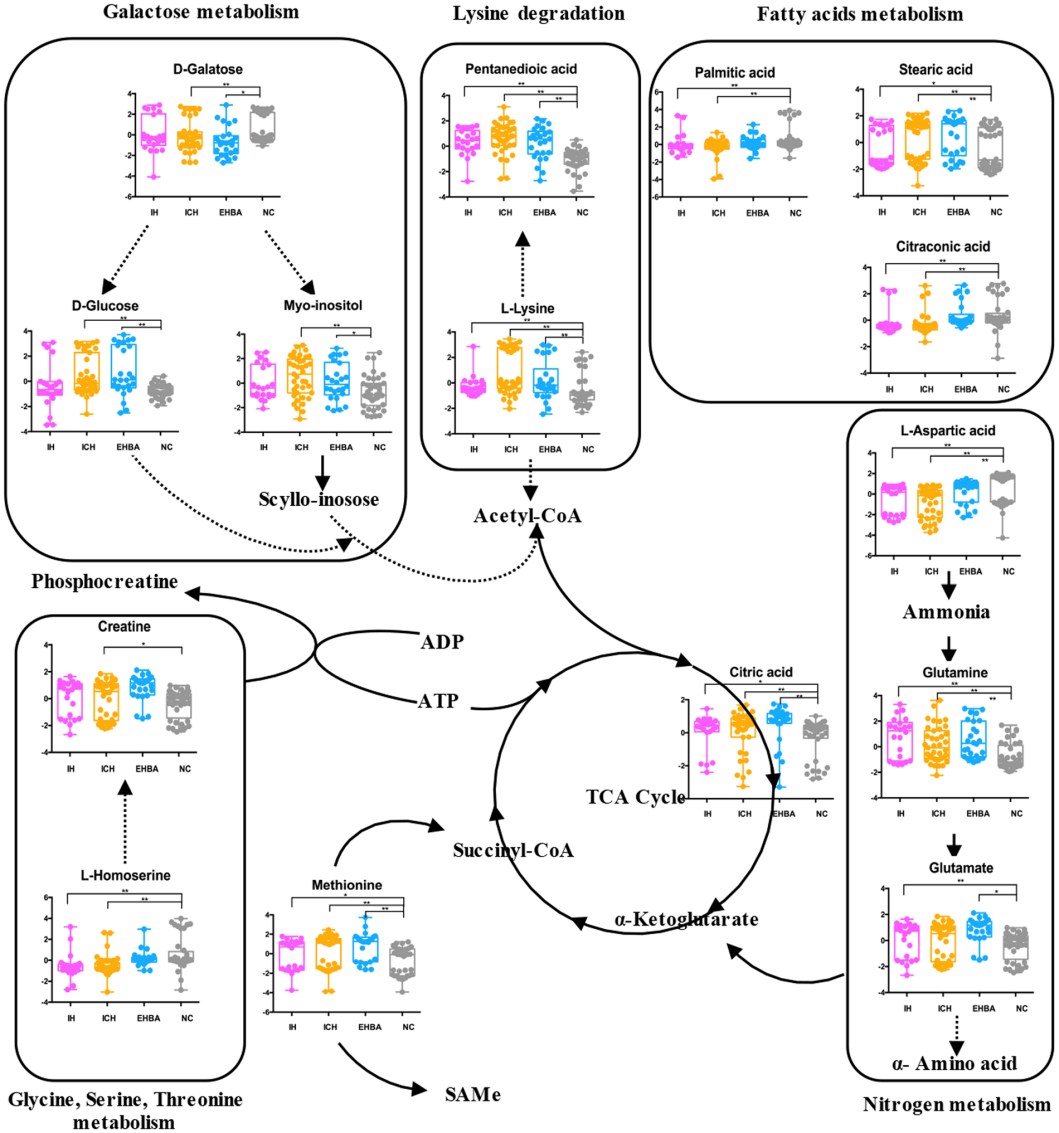
A simple diagram to illustrate the metabolites disturbance in HCMV induced liver injury infants. *P < 0.05, **P < 0.01.

In this study, the potential of metabolomics to identify biomarkers related to HCMV-induced liver injury in the plasma of infants was investigated. We divided the patients into three subgroups based on their clinical manifestations and identified 29 differential metabolites involved in a variety of amino acid, fatty acid and energy metabolism disorders. A simple diagram to illustrate the metabolites disturbance is shown in Fig. 5. Furthermore, a set of 5 metabolites (carbamic acid, glutamate, L-aspartic acid, L-homoserine and noradrenaline) were significantly increased in HCMV EHBA infants, which may distinguish HCMV EHBA from HCMV ICH.

The increase in glutamine and glutamate metabolism during HCMV infection was previously demonstrated in a series of *in vitro* experiments^20,23,24^. In the present study, glutamine was significantly increased in all sub groups of patients, while glutamate was increased only in the HCMV EHBA group. HCMV increased glutamine metabolism because HCMV required glucose for use in other synthetic purposes, such as fatty acid synthesis, rather than metabolism in the tricarboxylic acid (TCA) cycle as energy. Thus, maintenance of the TCA cycle and ATP levels must be transferred to glutamine^22^. In previous studies, glutamine and glutamate dehydrogenase activities were reported to increase during HCMV infection, confirming our findings^23^. However, glutamate was reported to be decreased in a clinical metabolic study of primary HCMV infection during pregnancy^25^. We hypothesised that this result was related to the complex mechanism of the human body after HCMV infection, and thus was not completely consistent with the results from the *in vitro* cell model. Glutamate also played a central role in hepatic amino acid metabolism. Liver disease can cause protein synthesis disorders and affect the activities of enzymes participating in glutamine synthesis^26^. For example, a metabolic disorder of glutamine and glutamate pathway was reported in carbon tetrachloride induced acute hepatotoxicity and in patients with jaundice syndrome^27,28^. Notably, plasma glutamate levels in infants with EHBA were reported to be significantly higher than those in infants with ICH, which is consistent with the result of our study^29^. However,whether the glutamine and glutamate disorder was caused by HCMV infection or hepatocellular injury remains unclear.

Fatty acid (palmitic acid, stearic acid, and citraconic acid) metabolism disorders were also observed in infants with HCMV-induced liver injury. As an enveloped virus, HCMV requires fatty acids to form a membrane^30,31^. A series of studies showed that HCMV induces the expression of glucose transporter 4 and its translocation to the cell surface, resulting in the accumulation of cytoplasmic glucose and increased fatty acid biosynthesis^21,32^. Mass spectrometry-based fatty acid analysis showed that very long-chain fatty acids (VLCFAs) are substantially increased in the lipids of infected cells and that saturated forms of these fatty acids are selectively incorporated into the virus envelope^33^. In addition, the human genome encodes seven long chain fatty acids on elongase enzyme into VLCFAs, which is pivotal for HCMV infection^34^. Taken together, these results suggest that lipid metabolism is a useful therapeutic target for treating HCMV infection. However, because of the limitations of the GC-MS platform, VLCFAs were not detected in this study. According to the results of our study and those of previous HCMV-related clinical metabolic studies, although the content of total fatty acid increased, specific fatty acids showed an increasing or decreasing trend^35^. Thus, we hypothesised that HCMV up-regulated specific VLCFAs for incorporation into the viral envelope. Although fatty acid metabolism is not essential in mammals, it is not easy to estimate the side effects of blocking fatty acid metabolism^36^. In future studies, we will use a ultra-high-performance liquid chromatography-MS (UHPLC-MS) platform to identify the specific VLCFAs involved in HCMV infection and evaluate the possibility of blocking specific VLCFAs.

Additionally, changes in amino acids were detected in patients. Liver is the centre of amino acid metabolism, and thus liver diseases are inevitably accompanied by amino acid perturbations. Disorders in amino acid metabolism have been reported in many liver-related diseases, such as cirrhosis, hepatic encephalopathy, hepatitis B, and cholestasis^37–40^. In the present study, a total of ten amino acids or derivatives were found to be disordered in one or more disease groups. The concentration of methionine (MET) increased in both the HCMV ICH and HCMV EHBA groups. The plasma clearance of MET is known to be reduced in patients with liver cell dysfunction with reductions in the mRNA of the main enzymes involved in MET metabolism^41,42^. *S*-Adenosyl-L-methionine (SAMe) is a metabolite present in all living cells and plays a key metabolic role as a major donor of methyl groups. Normally, SAMe is synthesized by methionine and ATP through the action of SAMe synthetase. It can effectively supplement glutathione in patients with liver disease and is widely used for treating liver disease (including cholestasis, hepatitis, and cirrhosis)^43^. Therefore, considering the presence of hepatocyte injury in the two subgroups, we orally administered SAMe to the infants. Interestingly, ingestion of exogenous SAMe may increase level of noradrenaline and vanillylmandelic acid in these two subgroups. Previous studies confirmed that SAMe can be used to promote the synthesis of monoamine neurotransmitters such as dopamine, noradrenaline, and 5-HT in the treatment of various nervous system diseases such as depression^44,45^. We hypothesised that this biological process led to elevated levels of noradrenaline and its metabolite vanillylmandelic acid in the HCMV ICH and HCMV EHBA groups.

Lysine biosynthesis and degradation are also involved in the process of HCMV induced liver injury. L-lysine and pentanedioic acid were up-regulated, while L-aspartic acid and L-homoserine were down-regulated in all three patient groups. As indicated in Table 1, infants in the three groups showed different levels of AST elevation. This abnormal increase in AST accelerated lysine consumption and produced oxaloacetic acid. Lysine is an essential amino acid, and elevated L-lysine levels in the plasma have been reported in both ICH and EHBA, which is consistent with our results^29,46^. Decreased concentrations of carnitine were also detected in the plasma. The data regarding carnitine in liver disease are controversial. The same results were found in the progress of primary biliary cirrhosis^47^. However, increased levels of carnitine and acylcarnitines have been observed in the plasma of patients with hepatic encephalopathy and cirrhosis^38,48^.

Finally, we also found that nitrogen metabolism in patient groups was affected to some degree. Ammonia is a degradation product of nitrogen compounds, which is metabolised into urea by the liver in healthy individuals. As shown in Table 1, blood ammonia increased to different degrees in all three subgroups. Typically, hyperammonemia occurs during liver diseases such as acute liver failure, hepatic encephalopathy and cirrhosis and reflects altered liver function or porto-systemic shunting^49,50^. However, the clinical treatment of reduced blood ammonia did not achieve satisfactory outcomes, prompting researchers to re-examine the correlation between blood ammonia and liver disease. For example, studies have shown that infection and systemic inflammation other than increased ammonia, are closely related to hepatic encephalopathy^51^. In addition, the downstream product of blood ammonia, α-ketoglutaramate, was found to be closely related to liver disease by affecting energy metabolism^52^. In the present study, the three metabolites (glutamate, citric acid, and d-galactose) showing specific changes only in the HCMV EHBA group were associated with energy metabolism. However, our present limited results showed that the TCA cycle in HCMV EHBA was up-regulated. Because of the limitations of our detection conditions, we did not detect other important intermediates in the TCA cycle, such as α-Ketoglutaramate. Energy metabolism of HCMV EHBA requires experimental confirmation.

To the best of our knowledge, this is the first study to explore different types of liver injury caused by HCMV. However, our study has several limitations: 1) The average age of children in this study was two months or more, and the lack of prior data made it difficult to trace whether these infants had primary HCMV infection, as saliva and urine polymerase chain reaction (PCR) tests are the gold standards for determining primary HCMV infection^53,54^. 2) The sample size was small and no prospective validation was conducted. We used all available samples for this study from specific period from Beijing Children’s Hospital. Because infants with HCMV infection are typically immunocompromised, often combined with other viruses or bacterial infection, these subjects were excluded from the study. Although our sample was small, the OPLS-DA model was stable and the differential metabolite screening process was rigorous. Thus, our results appear to be reliable. 3) Because of the characteristics of the GC-MS platform, we only detected small molecules with m/z values ranging from 50 to 500, making it difficult to comprehensively understand the overall metabolic process of liver injury caused by HCMV. In future studies, we will conduct UHPLC-MS to comprehensively evaluate metabolic changes in HCMV-induced liver injury in infants.

## Materials and Methods

### Study subjects

One hundred twenty-seven plasma samples were collected from HCMV IH patients (n = 22), HCMV ICH patients (n = 39), HCMV EHBA patients (n = 26), and sex- and age- matched healthy subjects (n = 40) recruited from Beijing Children’s Hospital between March 1, 2015 and March 1, 2017. The study was approved by the ethics committee of Beijing Children’s Hospital in accordance with the Declaration of Helsinki.

HCMV infection was defined as anti-HCMV–specific IgM positivity or increased anti-HCMV–specific IgG titres by more than 4-fold, and/or HCMV DNA positivity in blood and/or urine via PCR. HCMV IH was defined as elevated alanine aminotransferase > 50 U/L without cholestatic disease. HCMV ICH was defined as elevated alanine aminotransferase > 50 U/L combined with direct hyperbilirubinemia defined as direct bilirubin > 6.8 μM. EHBA was diagnosed by radionuclide hepatobiliary dynamic imaging and B ultrasonography.

The detailed medical history, physical examination, and biochemical profile of the study groups are shown in Table 1. The exclusion criteria for both patients and controls included hepatitis A–E virus and other hepatotrophic virus (Epstein-Barr virus, herpes viruses, adenoviruses, and parvovirus) infection. In addition, patients with concomitant acute or chronic severe diseases that may interfere with the evaluation of subjects(e.g., inborn errors of metabolism, genetic disease, cardiovascular disease, renal failure, diabetes mellitus, or any autoimmune disease) were also excluded from the study.

Fasting plasma was prepared within 1 h after blood withdrawal by centrifugation and stored at −80 °C until analysis. All examinations and experiments including plasma sample collection were carried out in accordance with relevant guidelines and regulations and were performed a er obtaining written informed consents.

### Chemicals and reagents

Methanol was obtained from Merck KGaA (Darmstadt, Germany). *N*,*O*-Bis (trimethylsilyl) trifluoroacetamide (BSTFA) with 1% trimethylchlorosilane (TMCS), Methoxyamine hydrochloride, pyridine, standard n-alkanes mixture (C8–C40), and 1,2-^13^C_2_-myristic acid were purchased from Sigma-Aldrich (St. Louis, MO, USA).

### Sample preparation and analysis

Each 50 μL aliquot of plasma was mixed with 200 μL methanol containing 50 μg 1,2-^13^C_2_-myristic acid as an internal standard.The mixture was vortexed for 3 min and centrifuged at 20,000 × *g* for 10 min. One hundred microliters of supernatant were dried in a SpeedVac sample concentrator at 45 °C for 2 h. The dried aliquots were combined with 30 μL methoxyamine hydrochloride in pyridine (10 mg/mL), then vortexed for 3 min and shaken at 30 °C for 90 min with the Thermo Mixer C (Eppendorf, Hamburg, Germany). Thirty microliters of BSTFA containing 1% TMCS were added to the sample and shaken at 37 °C for 30 min. The mixture was then transferred to a sampler vial with a glass insert and subjected to GC–MS analysis. QC samples were prepared by pooling aliquots of all the plasma samples and were processed with the same procedure as that for the experiment samples. During analyses of the sample sequence, one quality control sample was run after every 10 injections.

Analysis was performed on a Trace 1310 Gas Chromatograph equipped with an AS 1310 autosampler connected to a TSQ 8000 triple quadrupole mass spectrometer (Thermo Scientific, Waltham, MA, USA) as described previously by Xie *et al*.^55^. To detect and eliminate retention time shifts, the standard n-alkane mixture (C8–C40) was injected into the GC/MS during analysis of each batch of samples.

### Data processing and statistical analysis

Raw data acquired from Xcalibur 2.2 software (Thermo Scientific) were converted to “abf” format with the ABF converter (http://www.reifycs.com/AbfConverter/index.html). The MS-DIAL^56^ with Fiehn library was used for raw peak exaction, data baseline filtering and calibration, peak alignment, deconvolution analysis, and peak identification. The RI was calculated relative to the standard n-alkane mixture (C8–C40). Metabolites were identified by mass spectra matching against the reference spectra in the NIST 2014 standard database built-in Xcalibur 2.2 software.

The data normalization step was completed by the MetaboAnalyst 3.0 (http:// www.metaboanalyst.ca). Three types of data normalization protocols had been used to to reduce systematic variance and to improve the performance for downstream statistical analysis. The first one was “normalization by sum”, which means normalize each sample to a constant sum so that it is comparable to the other. “log transformations” was the second method to correct for heteroscedasticity, pseudo scaling and make multiplicative models additive. The former two methods were used for univariate analysis. The third method “pareto scaling” was used to reduce the relative importance of large values, but keep data structure partially intact^57,58^. Student’s *t* test, volcano plot and the heatmap analysis were also performed using MetaboAnalyst 3.0. SIMCA-P 13.0 (Umetrics, Umea, Sweden) was used to perform multivariate statistical analysis of OPLS-DA and obtain the variable influence on the projection (VIP) value of each metabolite. Significant metabolites were selected from the volcano plot with Log (fold-change) > 1 (or < -1), the Student’s *t* test **P** value threshold < 0.05 and VIP > 1 in the above OPLS-DA model.

## Electronic supplementary material

Supplementary Information
