# Supplementary material for: Disturbance in Plasma Metabolic Profile in Different Types of Human Cytomegalovirus-Induced Liver Injury in Infants
Source: Sci Rep. 2017 Nov 16;7:15696. doi: 10.1038/s41598-017-16051-8 (PMC5691185; doi:10.1038/s41598-017-16051-8)
Supplement: Supplementary file 1 — Supplementary Information [file 41598_2017_16051_MOESM1_ESM.pdf]

Supplementary Files

Disturbance in Plasma Metabolic Profile in Different Types of Human Cytomegalovirus-Induced Liver Injury in Infants

Wei-Wei Li[1,2], Jin-Jun Shan[1,2], Li-Li Lin[1,2], Tong Xie[1,2], Li-Li He[4], Yan Yang[*3]& Shou-Chuan Wang[1*]

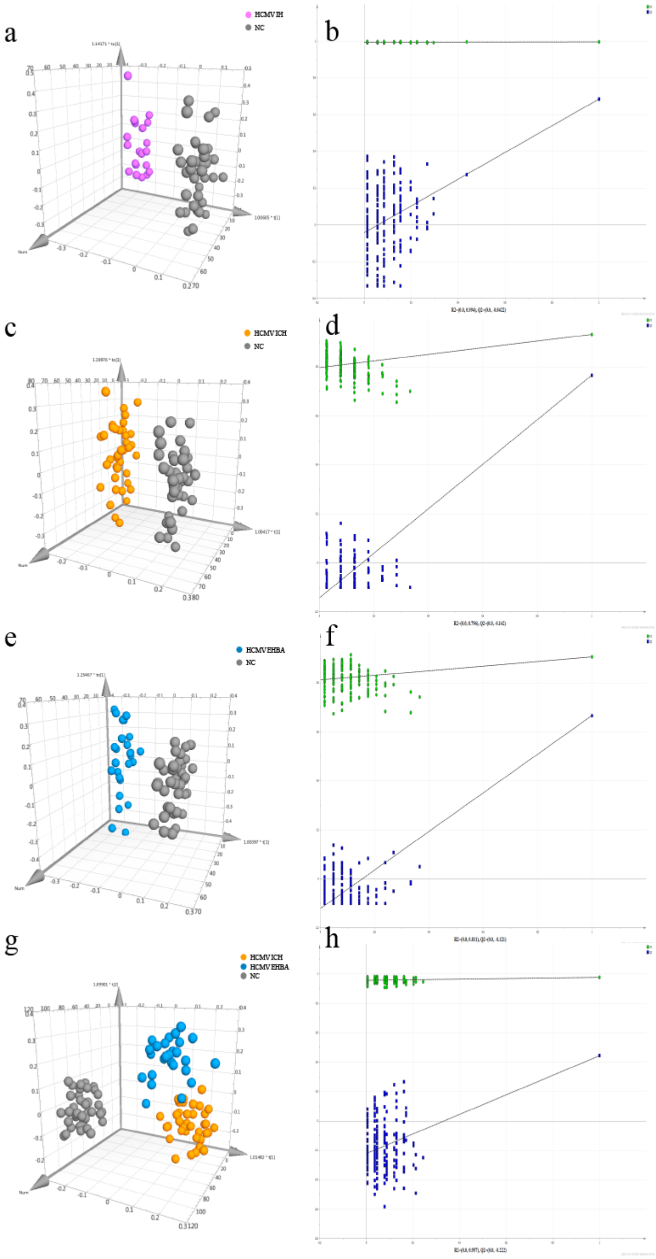

Supplementary Figure 1. OPLS-DA 3D score plots for discriminating the plasma metabolome from NC and HCMV induced liver injury subgroups obtained from GC-MS. (**a**) HCMV IH *vs* NC, (**c**) HCMV ICH *vs* NC, (**e**) HCMV EHBA *vs* NC, (**g**) HCMV ICH, HCMV EHBA *vs* NC. Chance permutation at 200 times was used for the discrimination between (**b**) HCMV IH *vs* NC, (**d**) HCMV ICH *vs* NC, (**f**) HCMV EHBA *vs* NC, (**h**) HCMV ICH, HCMV EHBA *vs* NC.

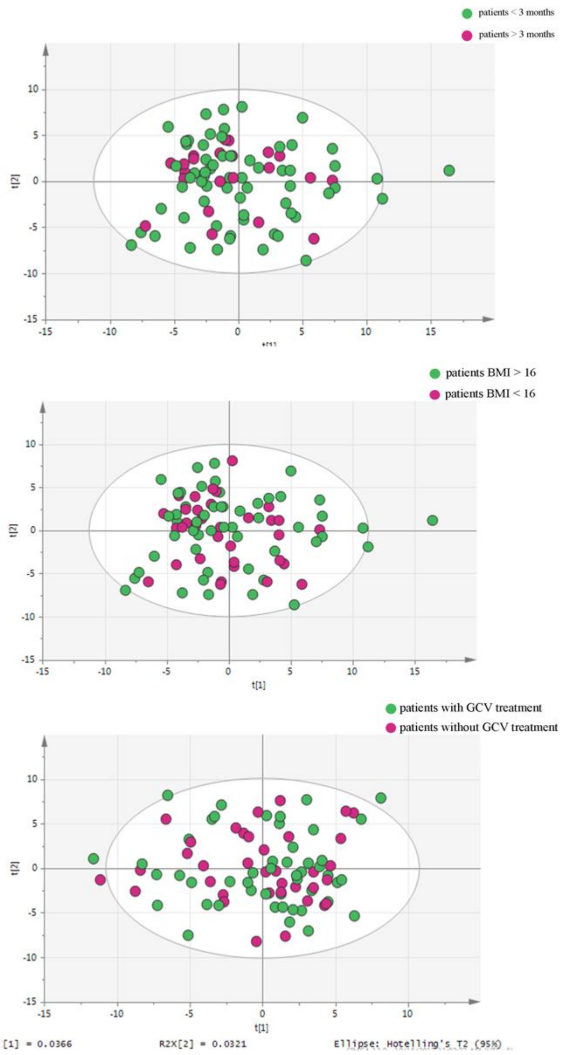

Supplementary Figure 2. (**a**) PCA Score plots shows no discrimination between patients >3 month and patients < 3 month, (**b**) PCA Score plots shows no discrimination between patients with BMI >16 and BMI < 16, (**c**) PCA Score plots shows no discrimination between patients with and without GCV use.

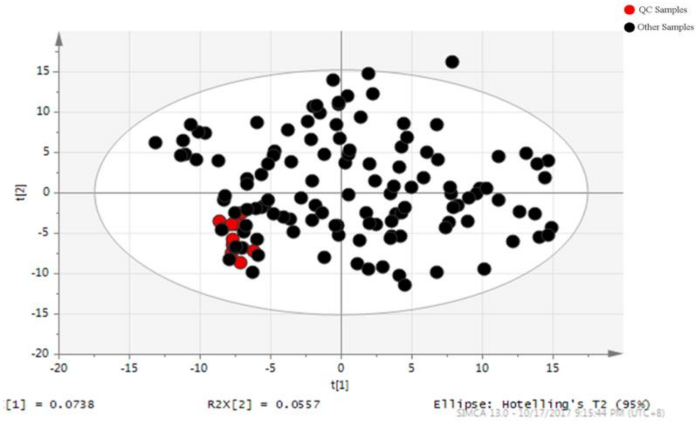

Supplementary Figure 3. PCA Score plots for the QC samples and the other samples

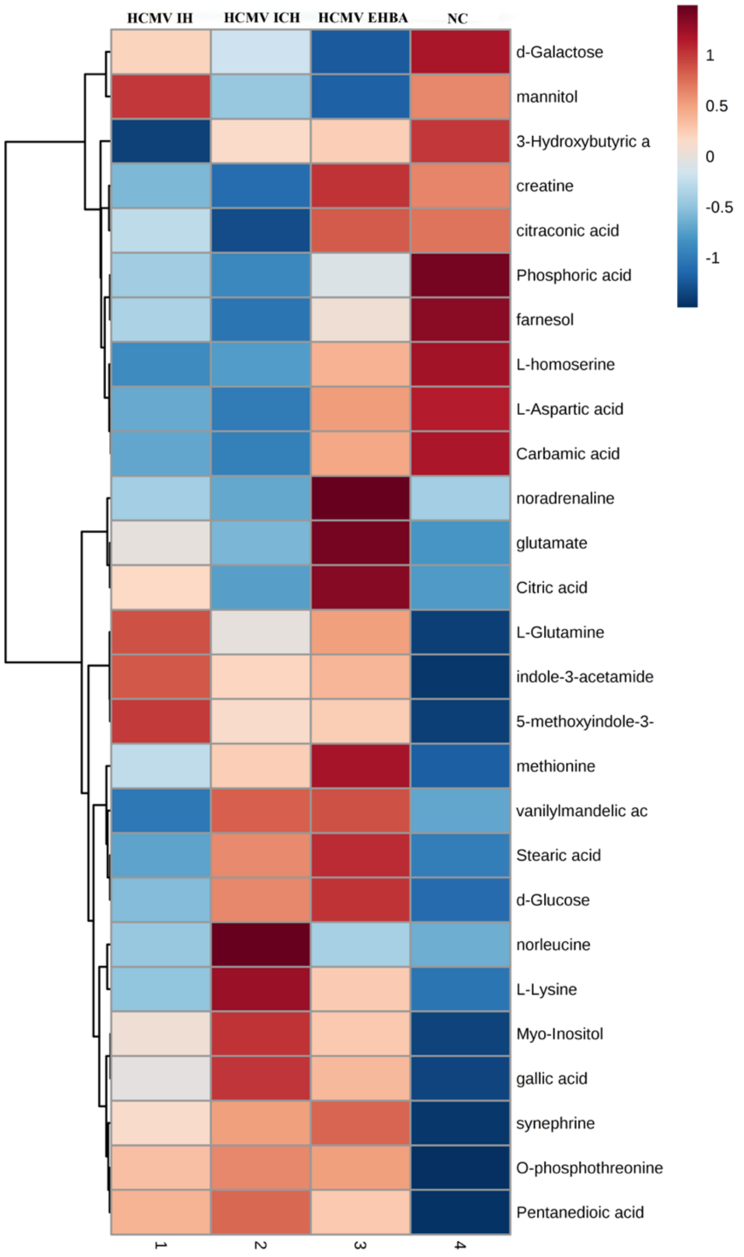

Supplementary Figure 4. Heatmaps visulization of the differential metabolites responding to HCMV IH, ICH, EHBA groups and NC.

**Model content**	**R^2Y**	**Q^2Y**	**R^2**	**Q^2**	**Identified metabolites**
HCMV IH VS NC	0.98	0.63	(0.0, 0.994)	(0.0, -0.042)	13
HCMV ICH VS NC	0.98	0.74	(0.0, 0.796)	(0.0, -0.142)	26
HCMV EHBA VS NC	0.99	0.67	(0.0, 0.811)	(0.0, -0.121)	17
HCMV ICH, EHBA VS NC	0.83	0.49	(0.0, 0.957)	(0.0, -0.222)	17

Supplementary Table 1. Summary of discrimination (OPLS-DA) modeling statistics for HCMV induced liver injury subgroups.

		HCMV IH *vs.* NC				HCMV ICH *vs.* NC				HCMV EHBA *vs.* NC			
**Variable**	**Description**	**VIP**	**LogFC**	**P value[a]**	**FDR**	**VIP**	**LogFC**	**P value[a]**	**FDR**	**VIP**	**LogFC**	**P value[a]**	**FDR**
3-hydroxy-L-proline	Amino acids	1.25	-0.42	<0.01	<0.01	**1.34**	**1.05**	**<0.01**	**<0.01**	0.31	-0.14	<0.05	0.18
Norleucine	Amino acids	0.86	-0.28	0.34	0.64	**1.67**	**2.62**	**<0.01**	**<0.05**	0.34	1.01	0.22	0.48
Creatine	Amino acids	1.13	-1.21	0.07	0.27	**1.50**	**-1.84**	**<0.01**	**<0.05**	0.34	-0.07	0.98	0.99
L-Lysine	Amino acids	**1.88**	**1.34**	**<0.01**	**<0.05**	**2.26**	**2.47**	**<0.01**	**<0.01**	**1.69**	**1.48**	**<0.01**	**<0.05**
L-Glutamine	Amino acids	**2.56**	**2.56**	**<0.01**	**<0.01**	**1.72**	**1.95**	**<0.01**	**<0.01**	**2.12**	**2.25**	**<0.01**	**<0.01**
L-Homoserine	Amino acids	**1.59**	**-1.65**	**<0.01**	**<0.01**	**1.64**	**-2.02**	**<0.01**	**<0.01**	0.76	-1.54	0.67	0.87
L-Aspartic acid	Amino acids	**2.02**	**-1.74**	**<0.01**	**<0.01**	**2.30**	**-2.10**	**<0.01**	**<0.01**	0.82	-0.90	<0.01	<0.01
Glutamate	Amino acids	0.95	0.89	<0.05	0.14	0.2	1.84	0.06	0.19	**2.34**	**1.62**	**<0.01**	**<0.01**
Methionine	Amino acids	1.15	1.15	<0.05	0.08	**1.47**	**1.77**	**<0.01**	**<0.01**	**2.36**	**2.71**	**<0.01**	**<0.01**
O-phosphothreonine	Amino acids	**2.15**	**2.22**	**<0.01**	**<0.01**	**2.36**	**2.61**	**<0.01**	**<0.01**	**1.67**	**2.66**	**<0.01**	**<0.01**
Pentanedioic acid	Fatty acids	**2.70**	**1.91**	**<0.01**	**<0.01**	**2.91**	**2.48**	**<0.01**	**<0.01**	**2.20**	**2.05**	**<0.01**	**<0.01**
Palmitic acid	Fatty acids	0.97	-0.92	<0.01	<0.05	**1.55**	**-2.31**	**<0.01**	**<0.05**	1.09	-1.65	0.45	0.72
Stearic acid	Fatty acids	1.73	1.39	<0.05	0.14	**2.15**	**2.93**	**<0.01**	**<0.01**	**2.27**	**1.97**	**<0.01**	**<0.01**
Citraconic acid	Fatty acids	1.07	-0.77	<0.01	<0.01	**1.56**	**-1.28**	**<0.01**	**<0.01**	1.23	-1.12	0.06	0.24
d-Galactose	Monosaccharide	0.95	-0.06	0.19	0.46	1.32	0.66	<0.05	0.12	**2.04**	**-1.37**	**<0.01**	**<0.01**
d-Glucose	Monosaccharide	0.89	3.11	0.37	0.67	**2.26**	**3.68**	**<0.01**	**<0.01**	**2.65**	**4.51**	**<0.01**	**<0.01**
Noradrenaline	Phenols	0.00	0.24	0.99	0.99	**1.12**	**1.16**	**<0.01**	**<0.05**	**1.73**	**1.08**	**<0.01**	**<0.01**
Vanillylmandelic acid	Phenols	0.25	0.21	0.7	0.87	**1.12**	**1.16**	**<0.01**	**<0.05**	**1.15**	**1.13**	**<0.01**	**<0.05**
Mannitol	Sugar alcohol	0.29	-0.17	0.96	0.99	**1.05**	**-1.50**	**<0.01**	**<0.05**	**1.26**	**-1.21**	**<0.01**	**<0.01**
Myo-Inositol	Sugar alcohol	1.48	1.16	0.08	0.28	**1.97**	**1.93**	**<0.01**	**<0.01**	1.54	1.43	<0.05	0.14
Glutaric acid	Mineral acids	**2.00**	**-1.82**	**<0.01**	**<0.01**	**2.71**	**-2.31**	**<0.01**	**<0.01**	**1.86**	**-1.61**	**<0.01**	**<0.01**
Gallic acid	Benzoic acids	**1.03**	**1.17**	**<0.01**	**<0.05**	**1.70**	**1.42**	**<0.01**	**<0.01**	**1.17**	**1.33**	**<0.01**	**<0.05**
Farnesol	Prenol lipids	**1.33**	**-1.06**	**<0.01**	**<0.05**	**1.53**	**-1.18**	**<0.01**	**<0.01**	0.90	-0.49	<0.05	0.21
Synephrine	Amine	1.73	1.39	<0.05	0.14	**1.95**	**1.55**	**<0.01**	**<0.01**	**2.03**	**1.97**	**<0.01**	**<0.01**
Citric acid	Tricarboxylic acids	0.85	0.49	<0.05	0.19	0.02	1.63	<0.05	0.08	**1.51**	**1.31**	**<0.01**	**<0.01**
Carbamic acid	Carboxylic acids	**2.13**	**-1.21**	**<0.01**	**<0.01**	**2.24**	**-1.07**	**<0.01**	**<0.01**	0.88	-0.44	<0.01	<0.01
3-Hydroxybutyric acid	Hydroxy acids	**1.24**	**-1.47**	**<0.01**	**<0.05**	**2.05**	**-2.05**	**<0.01**	**<0.01**	0.39	-0.98	0.11	0.32
Indole-3-acetamide	Indoles	**2.27**	**1.41**	**<0.01**	**<0.01**	**1.49**	**1.28**	**<0.01**	**<0.05**	1.78	1.52	<0.01	0.06

5-methoxyindole-3-acetic acid	Indoles	**2.00**	**1.54**	**<0.01**	**<0.05**	**1.22**	**2.03**	**<0.01**	**<0.05**	1.81	1.14	<0.01	<0.01

Supplementary Table 2. Levels of significantly changed metabolites in HCMV induced liver injury subgroups compared to NC revealed by untargeted metabolomics analysis. [a] **P** value of independent samples *t*-test with FDR correction.

**Pathway Name**	**Match Status**	**P value[a]**	**-log(P)**	**Holm P**	**FDR[b]**	**Impact**
Lysine biosynthesis	3/32	0.0056	5.19	0.45	0.18	0.10
D-Glutamine and D-glutamate metabolism	2/11	0.0067	5.00	0.53	0.18	0.35
Nitrogen metabolism	3/39	0.0097	4.63	0.76	0.18	0.07
Aminoacyl-tRNA biosynthesis	4/75	0.0101	4.60	0.78	0.18	0.11
Galactose metabolism	3/41	0.0112	4.49	0.85	0.18	0.23
Glycine, serine and threonine metabolism	3/48	0.0172	4.06	1.00	0.23	0.07
Cysteine and methionine metabolism	3/56	0.0259	3.65	1.00	0.30	0.06
Alanine, aspartate and glutamate metabolism	2/24	0.0308	3.48	1.00	0.31	0.47

Supplementary Table 3. Metabolic pathways between HCMV induced liver injury infants and NC.

[a] **P** value is the original **P** value calculated from the enrichment analysis;

[b] FDR is the portion of false positives above the user-specified score threshold.

NAME	RT	P value[a]	FDR	Fisher's LSD[b]	VIP
Butyraldehyde	17.68	0.0022106	0.025595	1 - 3; 2 - 3	1.25
Carbamic acid	6.01	2.63E-06	0.00014765	2 - 1; 3 - 1	2.07
d-Galactose	10.17	0.0026293	0.027734	3 - 1; 3 - 2	1.51
d-Glucose	10.23	1.39E-05	0.00050048	1 - 3; 2 - 3	2.19
Glutamate	8.5	0.0022867	0.025662	2 - 1; 2 - 3	2.11
Glutamin	9.39	0.0051514	0.046454	3 - 1; 3 - 2	1.15
L-Aspartic acid	7.53	1.54E-05	0.0005185	2 - 1; 3 - 1	2.04
L-Glutamine	9.36	0.00019344	0.0039076	1 - 3; 2 - 3	1.74
L-Homoserine	7.44	0.00087869	0.013051	2 - 1; 3 - 1	1.46
L-Lysine	9.01	0.00030076	0.0050628	1 - 3; 2 - 3	1.75
Maleimide	4.81	0.0019969	0.02401	1 - 2; 1 - 3	1.33
Methionine	7.92	0.00029725	0.0050628	1 - 3; 2 - 3	1.98
Noradrenaline	6.2	0.00028127	0.0050628	2 - 1; 2 - 3	1.54
O-Phosphothreonine	11.15	0.00058779	0.008995	1 - 3; 2 - 3	1.56
Pentanedioic acid	8.22	1.28E-09	1.08E-07	1 - 3; 2 - 3	2.46
Synephrine	5.43	0.00016591	0.0036428	1 - 3; 2 - 3	1.88
Tagatose	10.01	0.0044502	0.041618	3 - 1; 3 - 2	1.21

Supplementary Table 4. Identification of significantly different potential endogenous metabolites in the HCMV ICH, HCMV EHBA and NC.

[a] **P** value of one way ANOVA

[b]1-HCMV ICH group, 2-HCMV EHBA group, 3-NC

	ALT	AST	TBIL	DBIL	TBA	GGT	ALP	LDH	PT	PLT	WBC	NH3
3-hydroxy-L-proline	0.066	0.082	0.154	0.167	-0.105	-0.066	-0.011	-0.002	0.180	-0.042	0.010	0.021
norleucine	0.018	0.061	0.055	0.192	0.124	0.042	-0.003	0.222	0.140	0.020	0.362	-0.053
creatine	0.167	0.167	0.207	0.192	0.146	0.096	-0.062	0.040	0.162	-0.182	0.041	0.100
L-Lysine	-0.021	0.079	0.257	0.255	0.044	-0.163	0.096	-0.061	0.128	-0.019	-0.083	-0.012
L-Glutamine	-0.080	0.190	0.186	0.140	-0.020	0.118	0.136	0.153	-0.092	0.039	0.043	0.099
L-homoserine	0.048	0.102	0.130	0.159	0.173	0.111	0.092	0.167	0.110	0.009	0.132	-0.018
L-Aspartic acid	0.201	0.224	0.214	0.207	-0.035	0.083	0.088	-0.104	0.062	-0.243	-0.143	0.100
glutamate	-0.012	-0.083	-0.114	-0.097	-0.296	0.008	0.063	0.046	-0.167	0.088	0.051	0.081
methionine	0.145	0.172	0.113	0.112	-0.033	0.032	-0.043	0.027	0.271	-0.143	-0.068	0.038
O-phosphothreonine	-0.074	-0.052	-0.058	-0.047	0.067	-0.038	-0.055	-0.038	-0.052	-0.068	-0.182	0.062
Pentanedioic acid	0.109	0.170	0.031	0.003	-0.205	-0.261	0.043	-0.082	-0.279	-0.045	-0.182	0.198
Palmitic acid	-0.055	-0.090	-0.182	-0.183	0.055	-0.136	-0.158	-0.058	-0.169	0.038	0.034	-0.015
Stearic acid	0.144	0.243	0.135	0.151	-0.071	-0.025	0.158	0.127	-0.015	0.064	0.042	0.224
citraconic acid	-0.008	-0.007	-0.053	-0.068	0.040	-0.012	-0.352	-0.061	0.050	0.087	-0.032	-0.068
d-Galactose	-0.086	-0.038	-0.015	-0.016	-0.095	-0.081	-0.050	-0.077	0.121	0.111	-0.071	-0.168
d-Glucose	0.095	0.104	0.255	0.271	0.083	0.147	0.060	-0.074	0.072	0.014	-0.123	0.067
noradrenaline	0.192	0.170	0.165	0.193	0.069	0.114	0.011	0.222	0.140	0.020	0.362	-0.053
vanilylmandelic acid	0.167	0.190	0.186	0.140	-0.020	0.118	0.136	0.153	-0.092	0.039	0.043	0.099
mannitol	-0.102	-0.243	-0.221	-0.194	-0.172	-0.132	-0.130	-0.442	-0.258	0.146	-0.342	-0.145
Myo-Inositol	-0.151	-0.027	0.072	0.096	0.053	-0.057	0.005	-0.080	-0.065	-0.012	-0.178	0.097
Phosphoric acid	-0.009	0.102	0.065	0.106	-0.108	0.043	-0.170	0.189	0.075	0.069	0.091	0.187
gallic acid	-0.141	-0.026	0.014	0.008	0.064	-0.074	-0.021	0.067	0.147	-0.029	-0.002	-0.090

**farnesol**	**0.070**	**0.151**	**0.105**	**0.075**	**0.047**	**0.153**	**0.031**	**0.149**	**-0.037**	**-0.025**	**0.128**	**-0.018**
**synephrine**	**-0.056**	**0.153**	**0.026**	**0.039**	**0.009**	**-0.086**	**-0.032**	**-0.108**	**0.147**	**-0.017**	**-0.123**	**-0.101**
**Citric acid**	**0.076**	**0.033**	**-0.090**	**-0.126**	**-0.430**	**-0.193**	**0.078**	**0.062**	**-0.031**	**-0.012**	**-0.010**	**0.041**
**Carbamic acid**	**0.082**	**0.134**	**0.147**	**0.165**	**0.107**	**0.092**	**0.084**	**0.134**	**0.071**	**0.110**	**0.121**	**0.055**
**3-Hydroxybutyric acid**	**-0.027**	**-0.133**	**0.150**	**0.153**	**-0.005**	**-0.166**	**0.216**	**-0.296**	**-0.041**	**0.049**	**-0.224**	**0.058**
**indole-3-acetamide**	**-0.053**	**-0.026**	**-0.123**	**-0.114**	**0.033**	**-0.108**	**0.018**	**-0.077**	**0.045**	**-0.074**	**-0.006**	**-0.246**
**5-methoxyindole-3-acetic acid**	**-0.068**	**-0.071**	**-0.058**	**-0.053**	**-0.329**	**-0.192**	**-0.003**	**-0.012**	**-0.064**	**0.074**	**-0.035**	**0.096**

Supplementary Table 5. Pearson correlation coefficient Yellow: positive correlation and **P** < 0.05, Green: negative correlation and **P** < 0.05

**HCMV IH *vs.* HCMV ICH**	**Clinical indicators**	**AUC (95% CI)**	**Sensitivity**	**Specificity**
	ALP	0.90 (0.81-0.99)	0.97	0.73
	TBA	0.92 (0.83-0.99)	0.87	0.77
**HCMV IH *vs.* HCMV EHBA**	AST	0.85 (0.77-0.98)	0.88	0.77
	TBIL	1.00 (1.00-1.00)	1.00	1.00
	DBIL	1.00 (1.00-1.00)	1.00	1.00
	TBA	0.95 (0.88-1.00)	0.96	0.91
	ALP	0.80 (0.66-0.93)	0.88	0.73
**HCMV ICH *vs.* HCMV EHBA**	TBIL	0.68 (0.55-0.82)	0.76	0.51
	DBIL	0.72 (0.60-0.85)	0.80	0.67
	TBA	0.67 (0.53-0.80)	0.64	0.67
	**Metabolites**	**AUC (95% CI)**	**Sensitivity**	**Specificity**
	Carbamic acid	0.83 (0.72-0.94)	0.89	0.67
	Glutamate	0.70 (0.57-0.82)	0.89	0.44
	L-Aspartic acid	0.78 (0.65-0.90)	0.69	0.85
	L-homoserine	0.79 (0.67-0.91)	0.85	0.77
	Noradrenaline	0.74 (0.64-0.87)	0.62	0.77
	All metabolites	0.86 (0.77-0.95)	0.73	0.87

Supplementary Table 6. Receiver operating characteristics (ROC) curves with area under curve (AUC) of clinical indicators and metabolites to discriminate HCMV IH, HCMV ICH patients from HCMV EHBA patients
